# Analyzing public demands on China’s online government inquiry platform: A BERTopic-Based topic modeling study

**DOI:** 10.1371/journal.pone.0296855

**Published:** 2024-02-15

**Authors:** Zhuoyuan Tang, Xuan Pan, Zhouyi Gu

**Affiliations:** 1 School of Information Technology, Zhejiang Financial College, Hangzhou, China; 2 Medical Record, Zhejiang University School of Medicine Sir Run Run Shaw Hospital, Hangzhou, China; Hosei University: Hosei Daigaku, JAPAN

## Abstract

This study aims to enhance governmental decision-making by leveraging advanced topic modeling algorithms to analyze public letters on the "People Call Me" online government inquiry platform in Zhejiang Province, China. Employing advanced web scraping techniques, we collected publicly available letter data from Hangzhou City between June 2022 and May 2023. Initial descriptive statistical analyses and text mining were conducted, followed by topic modeling using the BERTopic algorithm. Our findings indicate that public demands are chiefly focused on livelihood security and rights protection, and these demands exhibit a diversity of characteristics. Furthermore, the public’s response to significant emergency events demonstrates both sensitivity and deep concern, underlining its pivotal role in government emergency management. This research not only provides a comprehensive landscape of public demands but also validates the efficacy of the BERTopic algorithm for extracting such demands, thereby offering valuable insights to bolster the government’s agility and resilience in emergency responses, enhance public services, and modernize social governance.

## Introduction

The "Online Government Inquiry Platform" refers to a digital interface established by government agencies to facilitate a two-way communication channel [1]. Through this platform, the public can submit inquiries, express concerns, and offer suggestions, while the government can respond, provide information, and demonstrate transparency in its operations and decision-making. In recent years, with the rise of emerging information technologies, online government inquiry platforms have seen rapid development and become an essential channel for reflecting social conditions, responding to public concerns, promoting problem-solving, and enhancing government governance capabilities. The public is increasingly partaking in online government participation by exercising democratic rights and expressing their preferences and opinions to the government through new media such as Weibo and WeChat, online forums, government websites, and other "online platforms" [2–4]. Public demands data, as a real-time source of social perception information, have the advantages of timeliness, high authenticity, and broad coverage. It can gather the issues faced by the public in a real-time, truthful, and comprehensive manner, thereby providing a basis for decision-making for government departments. Consequently, public demands have experienced explosive growth. According to statistics from the Shanghai Municipal Letters and Calls Bureau, a total of more than 69,000 public opinions and suggestions were received and handled in 2021, with a monthly average of 5,750, and an average public response rate of 79.08% [5]. The "People Call Me" platform, launched by Zhejiang Province, serves as a unified government service platform where the public can engage in online consultations, report issues, and make suggestions on a variety of topics, including housing provident fund inquiries, subway noise complaints, and policy recommendations. This platform also transparently discloses how various departments and municipalities address these consultations, grievances, and suggestions. Since its inception in 2017, the platform has received a total of 3,502,036 correspondences, achieving a public satisfaction rate of 96.03%. With the vast amount of text information on the online government inquiry platform, China’s government departments face the challenge of determining how to efficiently identify categories of citizen demands, mine hotspots and common issues of citizen demands, and discover the evolution of citizen demands.

Numerous scholars have conducted research on public demands data from online government inquiry platforms. Das et al. [6] focused on the exponentially growing citizen demands, using text mining and sentiment analysis to identify priorities in demands processing, enabling ordinary citizens to obtain timely public services and government support. Wang et al. [7] used neural network algorithms to design a text classification-based automatic forwarding method for government website mailboxes, optimizing the operation of leadership mailboxes and reducing manpower and administrative costs. Lei and Liu [8] analyzed the characteristics of public demands and government responses in sudden public health incidents using artificial coding, sentiment analysis, and statistical methods. Hu et al. [9] applied LDA [10] modeling methods to mine text data from government leaders’ electronic mailboxes in various provinces, comparatively analyzing the provincial differences in social governance issues, providing a reference for constructing a panoramic view of social concerns and supporting government governance decisions. Li et al. [11, 12] through case study methods, found that a smart emergency system based on social demands data is initially forming. Social demands data can promote information integration and utilization in emergency decision-making, boosting efficiency. Zhang et al. [13] applied the idea of data storytelling to the analysis of public demands in government-people interaction data, providing a new perspective and research approach for government departments to efficiently discern demands from massive interaction data. Teng and Guo [14] used the Fuzzy Set Qualitative Comparative Analysis method (fs/QCA) to analyze the configuration of factors influencing local government responsiveness, proposing paths to optimize it. Chang and Zheng [15] analyzed the impact of government response on public participation, discovering that government responses can drive continuous public engagement.

In summary, previous academic research has primarily focused on government response efficiency, the refinement of operational mechanisms, and the active participation of citizens in the government inquiry process. While various methodologies including case studies, text mining, sentiment analysis, and LDA topic modeling have been employed to gain a deeper understanding of public demands, there is room for improvement in these methods, particularly when dealing with complex texts and discerning nuanced semantic distinctions. For instance, LDA topic modeling, despite its widespread adoption, might sometimes struggle with semantic interpretation and processing of short texts, as noted by Niu et al. [16] and Udupa et al. [17]. More concerningly, the analysis of topic evolution has rarely been touched upon in past research. Few studies have attempted to incorporate dynamic topic modeling into the demands analysis process, which to some extent has limited the government’s ability to understand and respond promptly to sudden public incidents.

In response to the aforementioned issues, this study employed web crawling technology to comprehensively collect public demands data from online government inquiry platforms. The analysis process first started from multiple perspectives, utilizing methods such as descriptive statistical analysis, keyword cloud display, public sentiment inclination analysis, and government response cycles to deeply scrutinize and discuss the public’s demands. Subsequently, this study introduced advanced technologies such as BERTopic topic modeling to conduct both static and dynamic topic mining on the collected data. This process not only helps accurately reveal the public’s genuine needs and intentions but also allows the timely discovery of potential social risks. Ultimately, this study aims to enhance the resilience and flexibility of the government in key areas of social governance, such as resource allocation, risk perception and management, and emergency decision-making, providing robust support for modernized governance.

## Related work

### Public demands in the realm of government affairs

The opinions collected by government departments serve not only as a reflection of the public’s core demands but also as crucial online feedback on various societal hot-button issues, playing a key role in assessing and enhancing government performance by aiding in the deep understanding and quantification of the public’s focus on these issues [18]. Lili [19] emphasizes the perception of citizen needs in the design and implementation of electronic public services in the absence of direct user feedback. Additionally, Li et al. [20] analyzed the demands for information resource sharing in Chinese urban government e-governance, noting that these needs stem from both functional requirements of the government and structural factors. Lankhorst and Derks [21] proposed a Service-Oriented Architecture focusing on creating demand-driven electronic services, highlighting the importance of cross-departmental collaboration. Thomas and Streib [22], along with Vykydalova [23], highlighted the significance of social media in enhancing local government participation in e-governance, revealing the potential of new media in government-citizen communication. Furthermore, an analysis conducted by Ahmed et al. [24] on the coverage of the Kartarpur Corridor by Indian and Pakistani English print media revealed how the print media shaped public perceptions of political issues through varied framing. This aspect was equally significant in understanding public needs in the domain of governance. During the COVID-19 crisis, the research conducted by Zhang and Yu [25], along with Tang et al. [26], delved into the dynamics between the supply of information by governments and the demands for such information from the public. These studies shed light on both the effectiveness and the challenges inherent in government communication strategies during public health emergencies. Research conducted by Hou et al. [27] and Zhang et al. [28] both focused on environmental policy, discussing how public demands for environmental quality influence environmental regulation and green technology innovation. In terms of accountability, research by Berliner et al. [29] revealed public demands for government transparency and accountability mechanisms, while Fan and Meng [30] demonstrated that effective governance mechanisms can enhance the quality and utilization of open data, strengthening government accountability. Ahangama [31] explored the role of social media in enhancing public participation and the accountability process. Research by Ji and Kim [32] focused on the public demands for regulatory intervention in the age of social media, analyzing how the public influences the formation of regulatory policies during corporate crises through social media. A study by Chatfield and Reddick [33] demonstrated the application of big data analysis in enhancing the agility of public services for customers, while Cheng et al. [34] used machine learning algorithms to predict public policies, showcasing the prospects of technology applications in the field of government affairs. These studies provide comprehensive insights into the analysis of public demands in government affairs. They highlight critical issues in e-governance, crisis communication, environmental policy, social media applications, and disaster recovery services, proposing practical solutions and methodologies. These insights significantly contribute to policy formulation and the enhancement of services in governmental sectors. Furthermore, they lay a solid research foundation, enriching academic discourse in these fields.

### Research related to topic modeling methods

Online government inquiry platforms and other social media have accumulated a large amount of unstructured text information, which traditional data collection and analysis methods struggle to handle. Researchers have turned to computational methods to collect and analyze these data. Topic modeling is a statistical technique used to identify underlying topics in a set of documents, representing these topics by the occurrence of words that constitute them [35]. Nowadays, an increasing number of social media data researchers are utilizing topic modeling to conduct text data mining and analysis. Topic modeling has been applied in various fields, including news, public health, urban planning, political science, and information systems [36]. In 2003, Blei et al. proposed probabilistic topic modeling methods represented by LDA, viewing topics as probability distributions of words and identifying topics related to document semantics by extracting word co-occurrence information at the document level, opening up a new direction for text mining research [37]. On this basis, a series of new topic models were proposed, including the Dynamic Topic Model (DTM) [38] to analyze topic hotspot changes, the On-Line LDA model [39] for incremental model updates, and the BTM [40] model more suitable for short text modeling. Although probabilistic topic models like LDA have been widely applied, they still face some challenges, such as the complex and time-consuming process of determining the number of topics, lack of intuitive interpretability in model results, sparsity issues when dealing with large-scale texts, and poor modeling effects for short texts. In recent years, with the development of deep learning technology, a series of neural network-based topic models have emerged, such as lda2vec [41], Top2Vec [42], and BERTopic [43]. Among them, the latest BERTopic algorithm has gradually taken a leading position in the field of topic modeling, having been applied by several researchers in different fields who have validated the superiority and adaptability of the BERTopic model through comparisons with other algorithms [44, 45].

## Materials and methods

**Fig 1:**
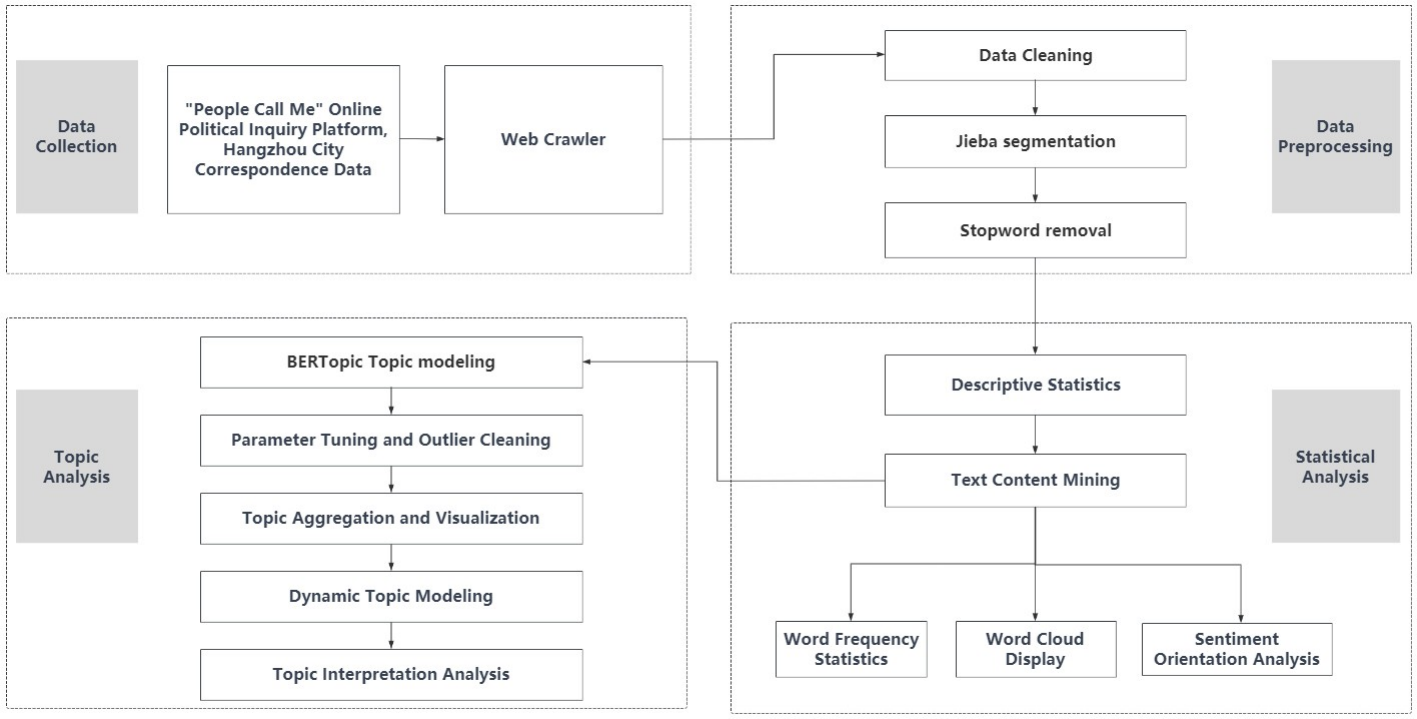
Research objectives flow.

The overall research process was divided into four steps (Fig 1): data collection, data preprocessing, statistical analysis, and topic modeling. In the statistical analysis phase, text content mining and descriptive statistical methods were used to conduct an exploratory analysis on the dataset, displaying the general information demands of the public on online government inquiry platforms from a macro perspective. The topic modeling phase focused on mining the features of public demands, revealing the characteristics of topic distribution and evolutionary trends.

### Data collection

This study selected the "People Call Me" unified platform in Zhejiang Province, China, as the data source. "People Call Me" is not only a significant reform innovation in Zhejiang Province’s digital transformation but also a model for the government’s digital platform, "holistic intelligent governance." Since the platform went online, it has integrated existing public opinion collection platforms in Hangzhou, such as the "12345 Mayor’s Hotline," "Public Sentiment Observation Room," and "Asking the People," creating a direct channel for public opinion. Together, they have woven a network for reflecting public sentiment, collecting social opinions, and managing grassroots society [46]. Using web crawler technology, 17,596 pieces of correspondence information from the Hangzhou area on the "People Call Me" website were crawled from June 2022 to May 2023. The data extracted included the title, content description, reflection time, reply opinions, reply units, and reply time.

### Data preprocessing

#### Data cleaning

Before initiating the detailed textual analysis, it was crucial to refine the dataset to ensure the reliability of the results. The data cleaning process began with the use of regular expressions for preliminary text preprocessing, which involved the removal of non-relevant characters, such as non-Chinese text, special symbols, and punctuation marks. Following this, the Python Pandas package was utilized for the removal of any blank entries and duplicates, streamlining the dataset by eliminating redundant data. Finally, through manual review, data that was incomplete or fell outside the predetermined time scope was excluded. Ultimately, a total of 17,593 pieces of data were included in the subsequent analysis (S1 File).

#### Jieba segmentation

We used Jieba for Chinese word segmentation. Jieba is an efficient Chinese word segmentation tool that can accurately divide sentences into individual words. Before segmentation, proprietary names such as "security certificate, public rental housing, no housing certificate, government services, Zhejiang Trade College, Zhejiang University City College, and community hospital" were added to Jieba’s custom dictionary to enhance the accuracy of the segmentation.

#### Stopword removal

In text analysis, the next crucial step after word segmentation is the removal of stop words to exclude the noise interference of related vocabulary and enhance the quality of the text. Stop words refer to words that frequently appear in the text but make no substantial contribution to understanding the semantics or themes of the text, such as "about," "the," "and," "oh," etc. Additionally, some polite expressions and titles, such as "hello," "leader," "hope," etc., have no practical significance for text analysis. In the process of constructing the stop word list, we adopted a comprehensive approach to effectively identify and remove stop words by merging multiple authoritative domestic stop word lists. These included the Chinese stop word list, Baidu stop word list, Harbin Institute of Technology stop word list, and the Sichuan University Machine Intelligence Laboratory stop word list, among others, to enhance the efficacy of removing stop words.

### Statistical analysis

The statistical analysis section was composed of two parts: text content mining and descriptive statistics. The text content mining part focused on extracting meaningful information from a large volume of correspondence data. By utilizing the Python Pandas package for word frequency statistics, and through the WordCloud [47] visualization method, these keywords could be intuitively presented, allowing researchers to quickly capture the public’s core demands and their distribution. Using the sentiment analysis tool in Baidu’s PaddleNLP [48], the emotional tendencies embedded in the public’s letters, such as positive, negative, or neutral, could be identified. This helped the government more accurately understand the public’s feelings and grasp the direction and intensity of public opinion. The descriptive statistics part aimed to understand the overall distribution and characteristics of the dataset, such as the proportion of each "reply unit" in the correspondence data, which could reveal the distribution of the public’s core demands; and the interval between "reply time" and "reflection time," which could provide insight into the speed and efficiency of the government’s response.

### Topic modeling

**Fig 2:**
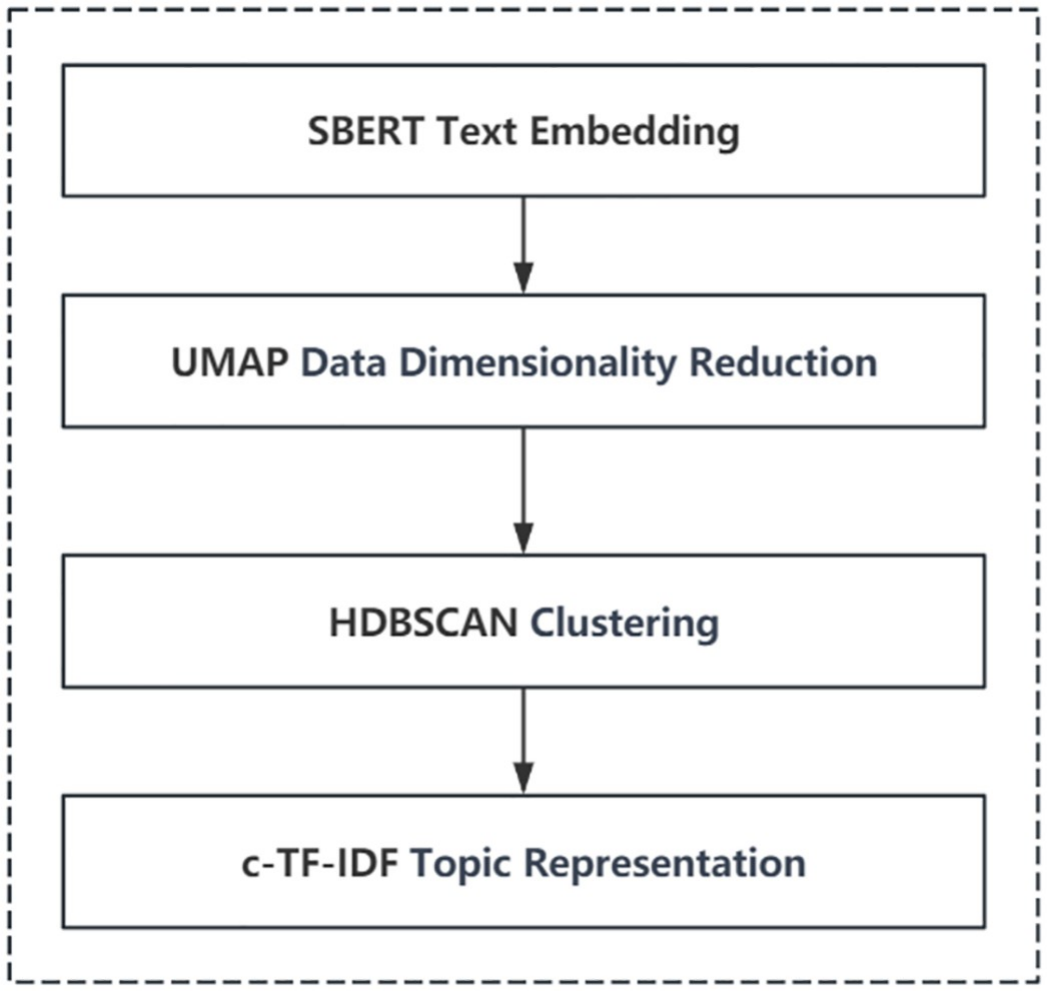
BERTopic workflow.

BERTopic is a topic modeling technique that utilizes Transformers [49] and c-TF-IDF to create dense clusters, thereby easily interpreting topics while retaining essential vocabulary in the topic description [43]. BERTopic supports a range of advanced topic modeling methods, including online topic modeling, dynamic topic modeling, supervised and semi-supervised topic modeling, and multimodal topic modeling, among others. BERTopic can be seen as a modular integration pipeline, primarily divided into four modules: text embedding, data dimensionality reduction, clustering, and topic representation, as shown in Fig 2. Each module supports various technical choices and can be seamlessly connected, allowing for significant flexibility in adjustments according to the experimental scenario and effects.

#### Text embedding

In the text embedding phase, BERTopic was responsible for converting the raw text into vector representations. This process supported various advanced embedding techniques, including SBERT [50], SpaCy, Scikit-learn, OpenAI, etc., and could utilize state-of-the-art pre-trained models to more accurately capture the semantic features of the text. The study selected "Paraphrase-multilingual-MiniLM-L12-v2" as the pre-trained model. Released by Sentence-Transformers, this model was a multilingual pre-trained model suitable for embedding Chinese text, containing 12 layers of Transformer structure, and had high performance with lower computational and storage resource requirements. The vectors obtained through text embedding captured the complex semantic information of the text, providing a solid foundation for various downstream tasks such as topic modeling, text classification, and text summary generation. This facilitated further analysis and interpretation.

#### Data dimensionality reduction

The high-dimensional vectors generated by SBERT text embedding could not be directly clustered due to the curse of dimensionality. One solution was to reduce the dimensionality of the high-dimensional vectors, and BERTopic supported dimensionality reduction methods such as PCA and UMAP. The default UMAP reduction method was capable of preserving the original semantic structure in a lower dimension as much as possible, enhancing the efficiency of processing large-scale text data while also improving the intuitive understanding and visualization capabilities of the topic model results [51].

#### Clustering

After the reduction in dimensionality, the vectors were to be clustered into similar embedding groups to extract our topics. BERTopic defaulted to using HDBSCAN [52] for clustering analysis, which was capable of capturing structures of different densities. Unlike methods that required the pre-setting of the number of topics, HDBSCAN could automatically discover clusters in the data and interpret each cluster as an independent topic [53]. This feature could save researchers a significant amount of effort previously spent on determining the number of topics, requiring only minor parameter adjustments based on experimental results.

#### Topic representation

After clustering was completed, BERTopic employed an improved method called c-TF-IDF to obtain precise representations of topics from the bag-of-words matrix. Unlike traditional methods that mainly focused on the importance of words in individual documents, c-TF-IDF emphasized the importance of words across the entire collection of topics. This approach helped to accurately reduce the number of topics to the user-specified quantity and enabled BERTopic to support various topic modeling methods, such as guided topic modeling, dynamic topic modeling, or class-based topic modeling [54].

### Data translation and validity assurance

In this study, all data in Chinese were translated into English for analysis purposes. Our experiments were based on the original Chinese data, and the results were translated to minimize semantic loss as much as possible, employing a two-stage method to ensure accuracy. Initially, a preliminary translation was performed using advanced translation software to provide a quick and rough text conversion. Subsequently, we engaged two bilingual experts to meticulously review the results of the software translation. These experts possess extensive experience in Chinese-English translation and have a deep understanding of the specialized terminology within our field of research. During the translation process, any uncertain or ambiguous translations were discussed by our research team to determine the most suitable English expressions. Moreover, to ensure the consistency and reliability of the translations, we employed a back-translation technique, where the translated texts were retranslated back into Chinese and compared with the original texts. Through this rigorous translation process, we strived to ensure the accurate conveyance of data semantics, thereby maintaining the validity of our research findings.

## Results

### Statistical analysis

**Fig 3:**
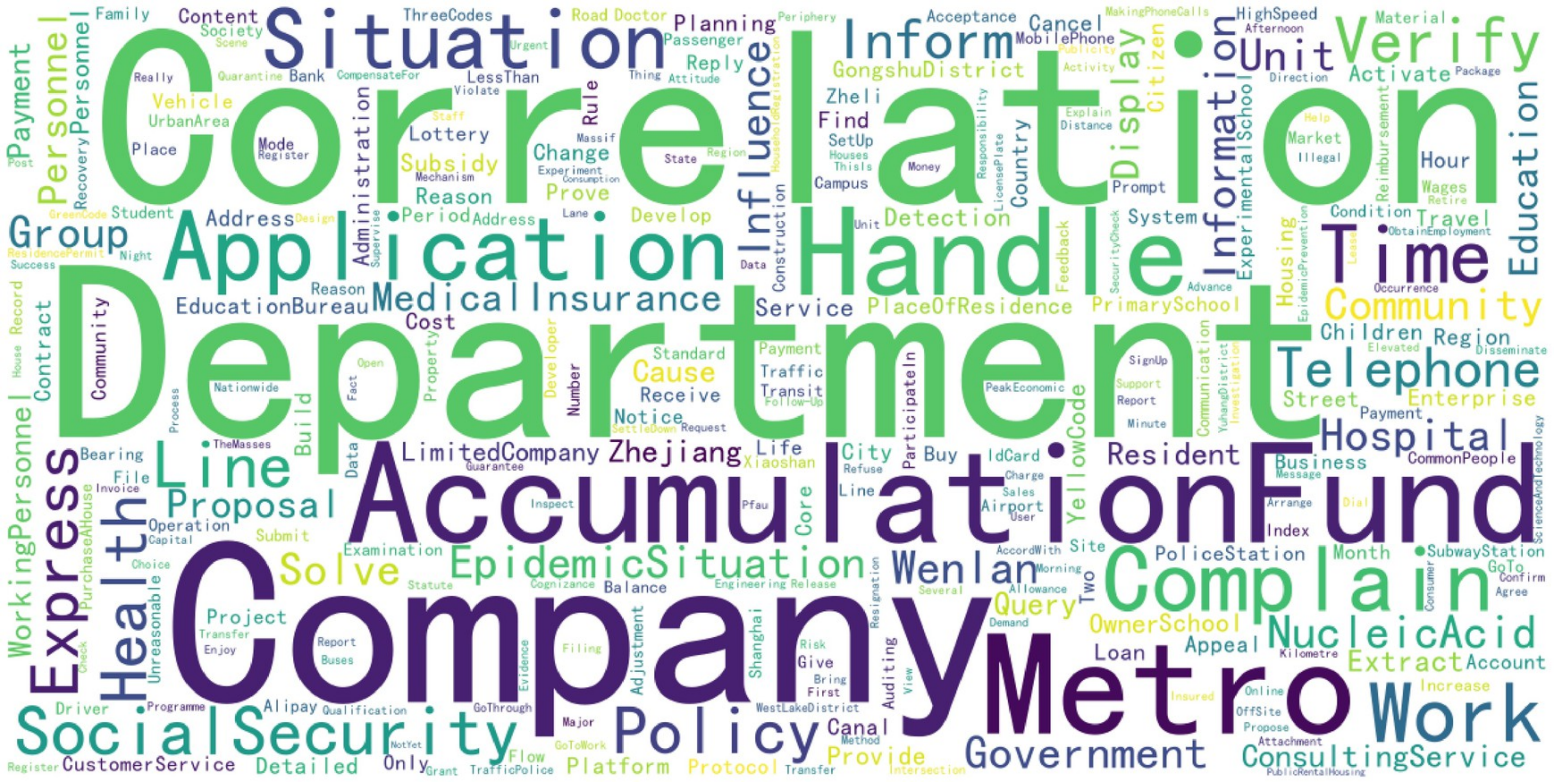
Word cloud of "Reflective Content" in letters.

Statistical results showed that Hangzhou received a total of 17,593 pieces of correspondence from June 2022 to May 2023, with an average of 48.2 received daily. Keywords were extracted from the content details of the issues presented by the citizens, and a word cloud (Fig 3) was used to display them, providing an intuitive representation of public demands. The word cloud was drawn based on the frequency of word occurrence, with larger words indicating higher frequency. From the Fig 3, it could be seen that the public’s demands were mainly concentrated in areas such as correlation, departments, accumulation fund, social security, metro, application, complain, and handle.

A frequency analysis was conducted on the detailed content of the issues reflected by citizens, and the results are shown in Table 1. Combining Fig 3 and Table 1, it can be seen that the issues articulated by citizens in the online correspondence are closely related to people’s daily lives, such as government policies, social security, transportation, public services, consumer rights, public health, education, etc.

**Table 1:** Frequency of keywords "Reflective Content" in letters.

Word	Frequency	Word	Frequency	Word	Frequency	Word	Frequency
Relevant	3794	Verify	1857	Health Insurance	1597	Talent	1153
Department	3773	Health	1838	Suggestion	1574	Subsidy	1094
Company	3259	Telephone	1805	Personnel	1545	Service	1093
Accumulation Fund	3184	Inform	1801	Display	1539	Provide	1077
metro	3169	Epidemic	1789	Education	1536	Detection	1071
Handle	2959	Nucleic Acid	1779	Consultation	1484	Yellow Code	1071
Application	2690	Wenlan	1746	Pay	1444	School	1053
Complaint	2584	Residential Area	1675	Staff	1394	Gongshu District	1046
Situation	2241	Hospital	1661	Residents	1391	Enterprise	1044
Time	2091	Government	1650	Zhejiang	1383	Travel	1041
Work	2067	Group	1638	Lead to	1376	Detailed	1038
Social Security	2044	Information	1635	Inquire	1341	Demand	1010
Express Delivery	1980	Resolve	1625	Withdraw	1250	Cancel	996
Line Number	1922	Unit	1600	Limited Company	1228	Reason	978
Policy	1916	Impact	1599	Property Owner	1196	City	977

**Fig 4:**
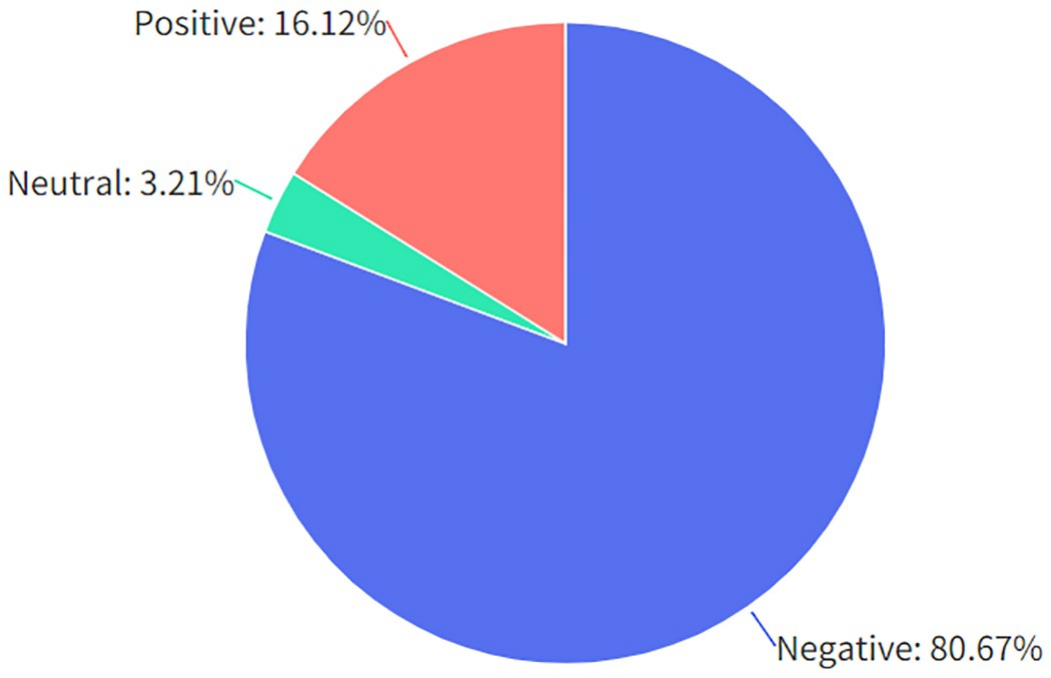
Results of the analysis of affective tendencies in letters.

An emotional tendency analysis was conducted on the details reflected in the letters, and the results are shown in Fig 4. Of the correspondents, 14,192 (80.67%) exhibited negative emotions, with complaints being the primary topic; 565 (3.21%) correspondents maintained a neutral attitude, with inquiries being the main topic; 2,836 (16.12%) correspondents held a positive attitude, with suggestions being the principal topic.

**Fig 5:**
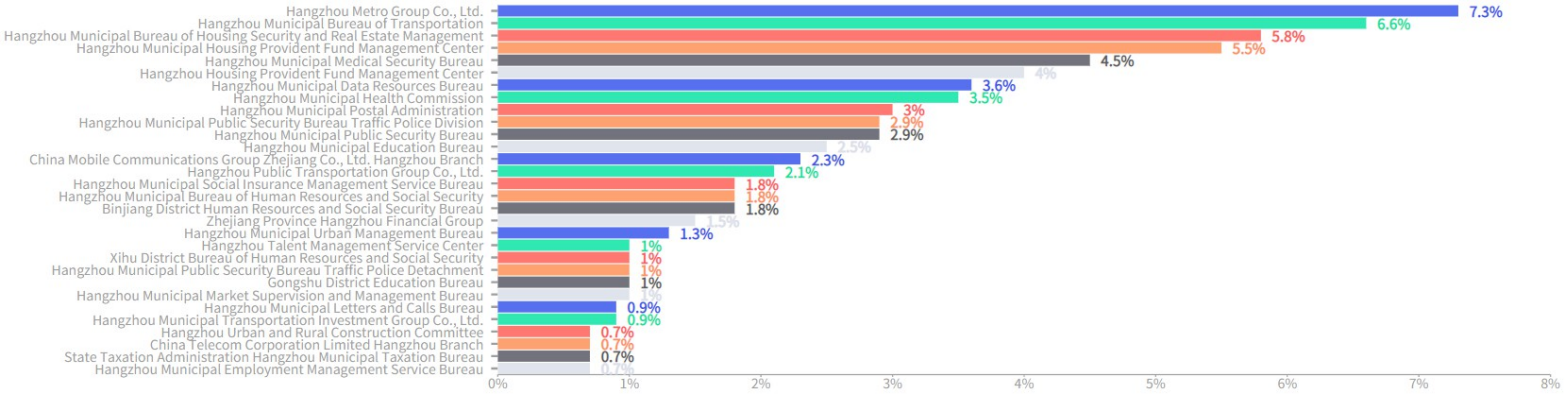
The distribution of the number of letters as a percentage among responding departments.

A statistical analysis was conducted on the accepting units in the letters, with 17,593 letters being allocated to 522 different units for processing and response. The number of letters received by each unit and the average response cycle are shown in Table 2, and the distribution of the number of letters by percentage is illustrated in Fig 5. It can be seen that Hangzhou Metro Group Co., Ltd. is the department that received the most letters, with a total of 1,281 (7.3%) letters. Despite bearing significant pressure, this department still maintained high efficiency in response, receiving an average of 3.5 letters per day, with an average response cycle of 4.06 days. Citizens’ attention mainly focused on departments related to transportation, housing, healthcare, accumulation fund, postal services, education, etc. These departments have an average response cycle of less than 10 days, indicating that they can actively and promptly respond to citizens’ questions and demands.

**Table 2:** The number of letters and the average response cycle of the responding departments(Top 30).

Reply Department	Number of Letters (copies)	Average Reply Cycle (days)	Reply Department	Number of Letters (copies)	Average Reply Cycle (days)
Hangzhou Metro Group Co., Ltd.	1281	4.06	Hangzhou Municipal Bureau of Human Resources and Social Security	317	2.31
Hangzhou Municipal Bureau of Transportation	1165	3.11	Binjiang District Human Resources and Social Security Bureau	313	3.85
Hangzhou Municipal Bureau of Housing Security and Real Estate Management	1021	4.38	Zhejiang Province Hangzhou Financial Group	256	3.02
Hangzhou Municipal Housing Provident Fund Management Center	959	1.56	Hangzhou Municipal Urban Management Bureau	221	5.30
Hangzhou Municipal Medical Security Bureau	792	3.37	Hangzhou Talent Management Service Center	184	2.92
Hangzhou Housing Provident Fund Management Center	711	1.05	Xihu District Bureau of Human Resources and Social Security	182	3.80
Hangzhou Municipal Data Resources Bureau	635	2.16	Hangzhou Municipal Public Security Bureau Traffic Police Detachment	174	2.39
Hangzhou Municipal Health Commission	613	2.65	Gongshu District Education Bureau	173	5.76
Hangzhou Municipal Postal Administration	532	8.30	Hangzhou Municipal Market Supervision and Management Bureau	172	2.26
Hangzhou Municipal Public Security Bureau Traffic Police Division	512	3.72	Hangzhou Municipal Letters and Calls Bureau	158	2.13
Hangzhou Municipal Public Security Bureau	507	2.96	Hangzhou Municipal Transportation Investment Group Co., Ltd.	151	6.23
Hangzhou Municipal Education Bureau	442	5.12	Hangzhou Urban and Rural Construction Committee	128	3.00
China Mobile Communications Group Zhejiang Co., Ltd. Hangzhou Branch	404	6.79	China Telecom Corporation Limited Hangzhou Branch	126	7.24
Hangzhou Public Transportation Group Co., Ltd.	372	7.03	State Taxation Administration Hangzhou Municipal Taxation Bureau	124	5.09
Hangzhou Municipal Social Insurance Management Service Bureau	322	2.96	Hangzhou Municipal Employment Management Service Bureau	123	2.17

**Fig 6:**
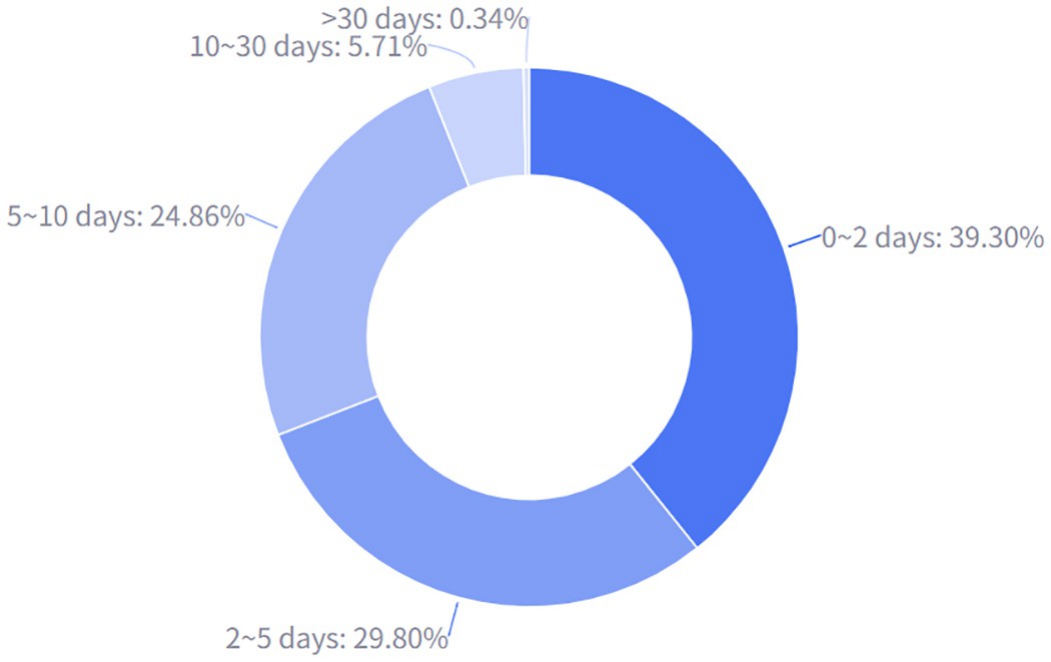
The distribution of the average response cycle among all departments.

A statistical analysis was conducted on the response cycles of all departments in the letters, and the results are shown in Fig 6. Of the 17,593 letters, 6,914 (39.3%) received timely responses within 2 days, indicating that many government departments are able to respond quickly to citizens’ demands The distribution of letters with response cycles of 2–5 days and 5–10 days was 5,242 (29.8%) and 4,374 (24.86%), respectively. These letters may involve more complex issues that require more time for communication and coordination. Letters with a response cycle of 10–30 days accounted for 5.71%, while those with a response cycle of more than 30 days accounted for only 0.34%. Although these percentages are relatively small, they are still worth attention. Such letters mainly involve judicial complaint-related events that require a longer time period for investigation and evidence collection, and there may be various difficulties in processing. Further in-depth analysis of these issues is needed to find ways to improve service efficiency. Such data also provide valuable information to government departments, helping to understand existing service efficiency and identify directions for improvement.

### Topic analysis

**Fig 7:**
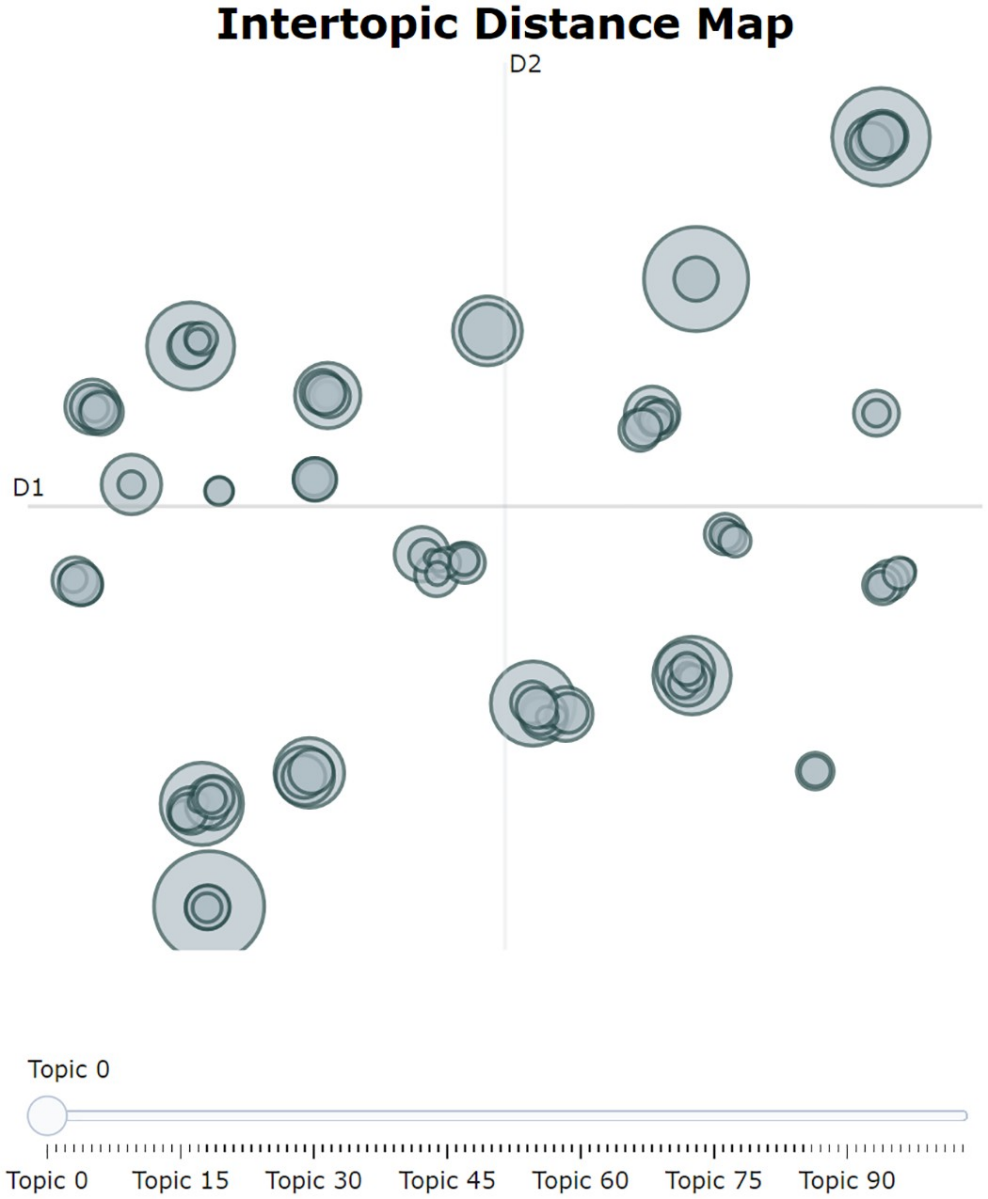
Fully automated generation of topic distributions. The figure illustrates the distribution of algorithmically-generated topics within a two-dimensional scaling space. Each circle in the chart represents an independent secondary topic, with the size of the circle generally reflecting the number of documents associated with that topic in the dataset. The placement of the circles indicates the relative distance and similarity between topics: those that are proximate to each other suggest similar thematic content, whereas circles that are more distant from each other indicate a larger disparity in content.

This study was based on the BERTopic algorithm, with the individual modules SBERT, UMAP, HDBSCAN, and c-TF-IDF selected for modeling. The initial modeling was done using default settings, incorporating the reduce_outliers algorithm to minimize noise interference. Without pre-setting the number of clustering topics, the model automatically generated 104 topics. From Fig 7, it can be seen that the overall topic distribution exhibits characteristics of small-scale aggregation and large-scale dispersion, suggesting that further aggregation can be achieved among small-scale subjects.

**Fig 8:**
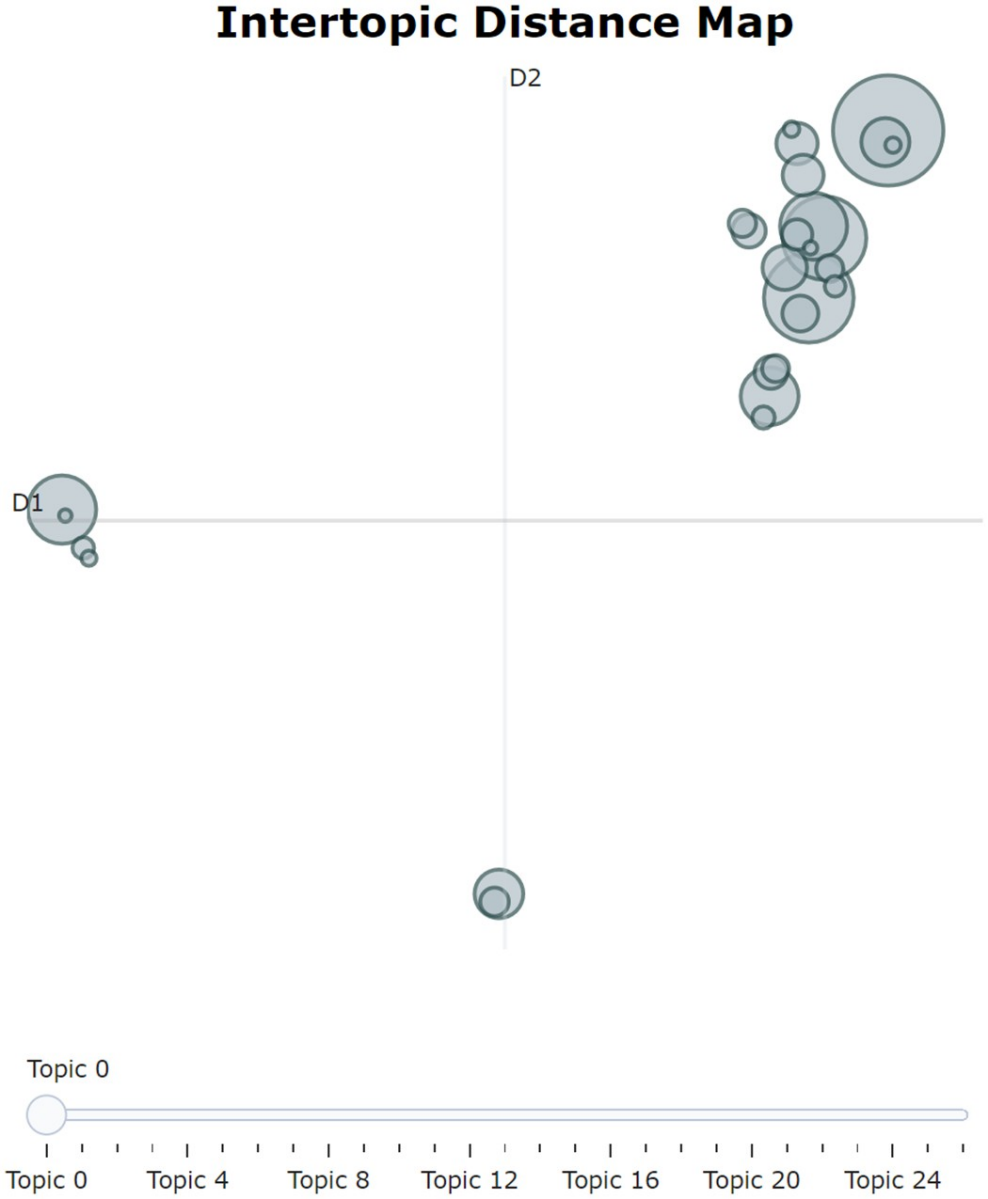
Secondary topic distribution diagram. The figure displays the distribution of secondary topics within a two-dimensional scaling space.

**Fig 9:**
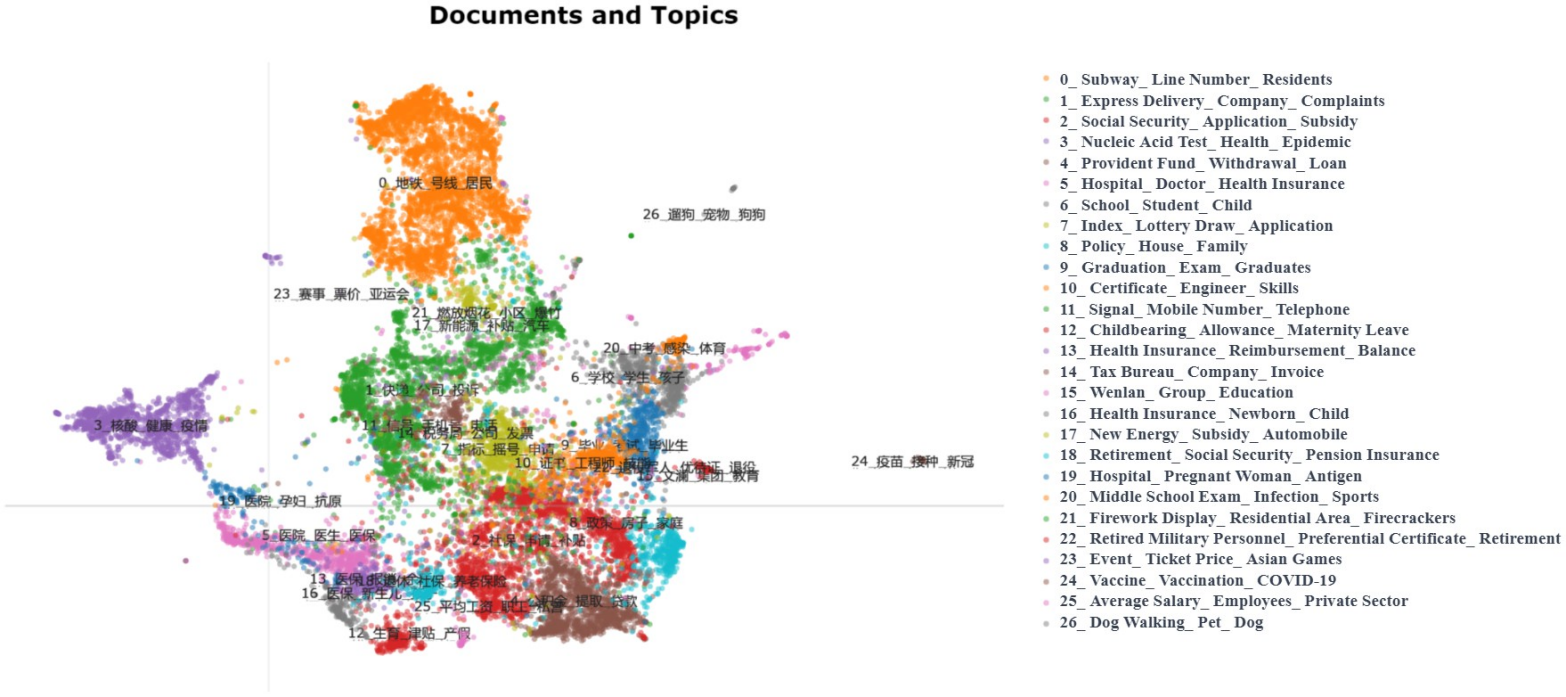
2D Distribution of secondary topic documents and their topics. The figure presents the distribution of secondary topic documents and their corresponding topics within a two-dimensional space, enabling rapid identification of the clustering of topics within the document set and the relative positioning of topics. This allows for the analysis of potential connections or differences between topics. Different colored blocks within the diagram represent distinct topics, with the list on the right detailing the specific content of each numbered topic. The spatial distribution of colored blocks signifies the degree of relatedness between document topics; blocks in close proximity indicate similar topic content, while those further apart suggest greater differences in topic content.

**Fig 10:**
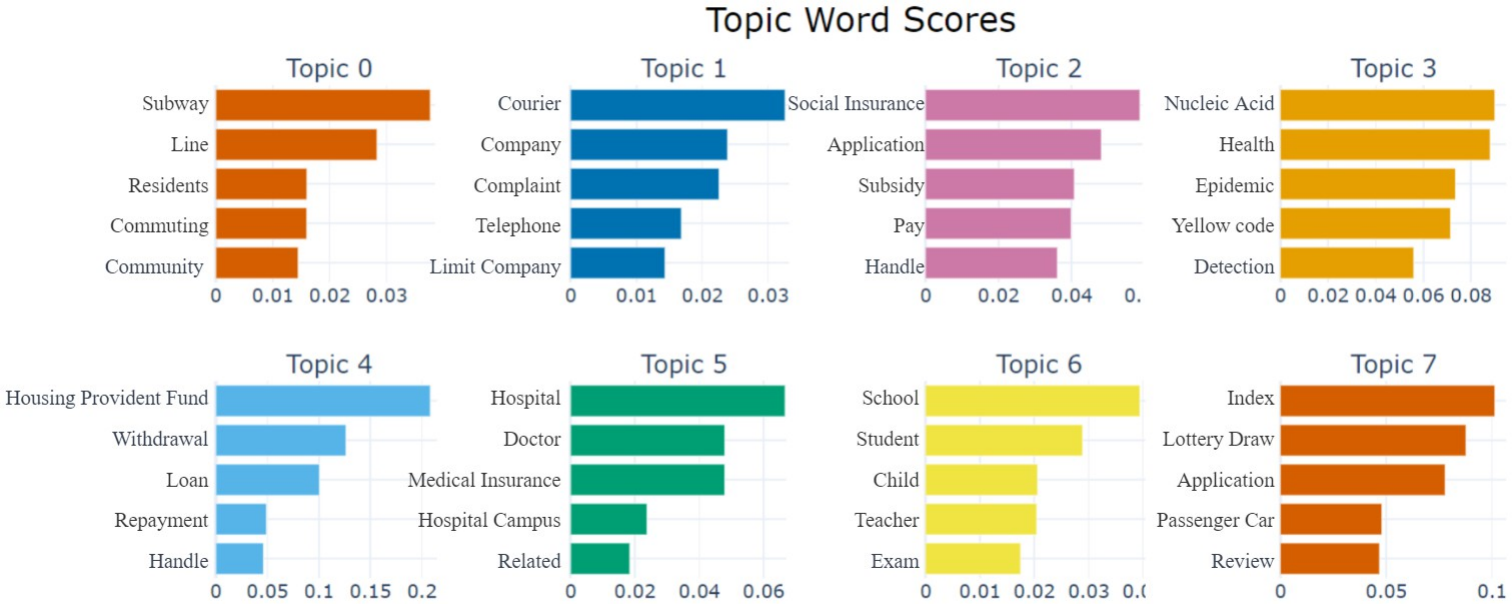
Secondary topic keyword contribution diagram. The figure illustrates the composition of different topics and identifies the most influential keywords defining these topics. Each subplot represents a topic, with the horizontal axis indicating the weight or contribution of keywords within the corresponding topic, and the vertical axis listing the keywords with the highest weight in each topic. The length of the bars in the bar graph represents the weight, with longer bars denoting a greater contribution of the keyword to the topic.

In the process of further determining the number of topics, we manually reviewed the original topic distribution in Fig 7 and the response unit situation in Table 2 to further optimize and aggregate the topics. By continuously adjusting the BERTopic-related parameters, such as min_topic_size, we finally determined that when the number of topics is 27, it can serve as the result for secondary topics. Fig 8 displays the distance distribution among the topics, and Fig 9 shows the distance distribution between the documents and their respective topics. In Figs 8 and 9, the closer the distance between topics or documents, the higher their similarity. Fig 10 displays the top 8 topics and the keywords with the highest c-TF-IDF scores within each topic.

**Fig 11:**
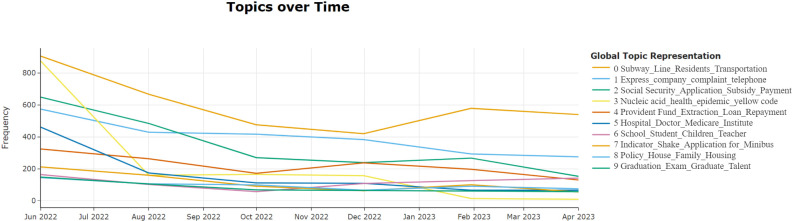
Secondary topic dynamic topic diagram. The figure illustrates the temporal trends of various topics. Different colors within the chart represent distinct topics, each detailed with a number and description in “Global Topic Representation” on the right. The horizontal axis denotes the time span, and the vertical axis represents the frequency, indicating the number of occurrences of each topic within the dataset.

Using the BERTopic dynamic topic modeling method, the evolution of the top 10 topics during the research period was visualized (Fig 11). From Fig 11, it can be seen that Topic 3 is related to epidemic prevention and control. With the adjustment and opening of China’s epidemic control policies at the end of 2022, this topic has gradually faded from the public’s view. The remaining topics overall maintain a stable trend, with the number of letters in 2023 slightly reduced compared to 2022.

**Fig 12:**
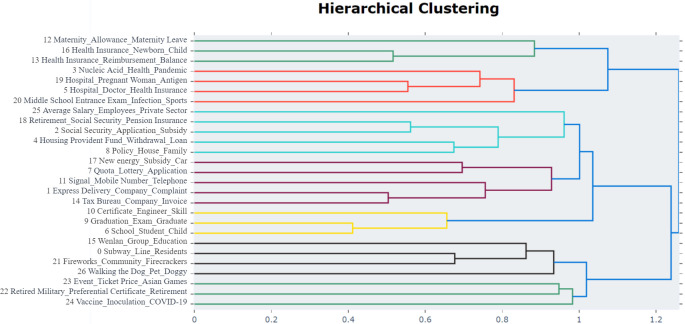
Secondary topic hierarchy diagram. The figure displays the relative relationships and hierarchical structure among extracted secondary topics. Each topic is labeled on the left side of the diagram with a number and a descriptor, while different colored lines represent various topic categories and clustering branches, aiding in the distinction of broader group relationships between topics. On the horizontal axis, smaller values indicate greater similarity in content between connected topics; larger values denote greater divergence among the topics.

**Fig 13:**
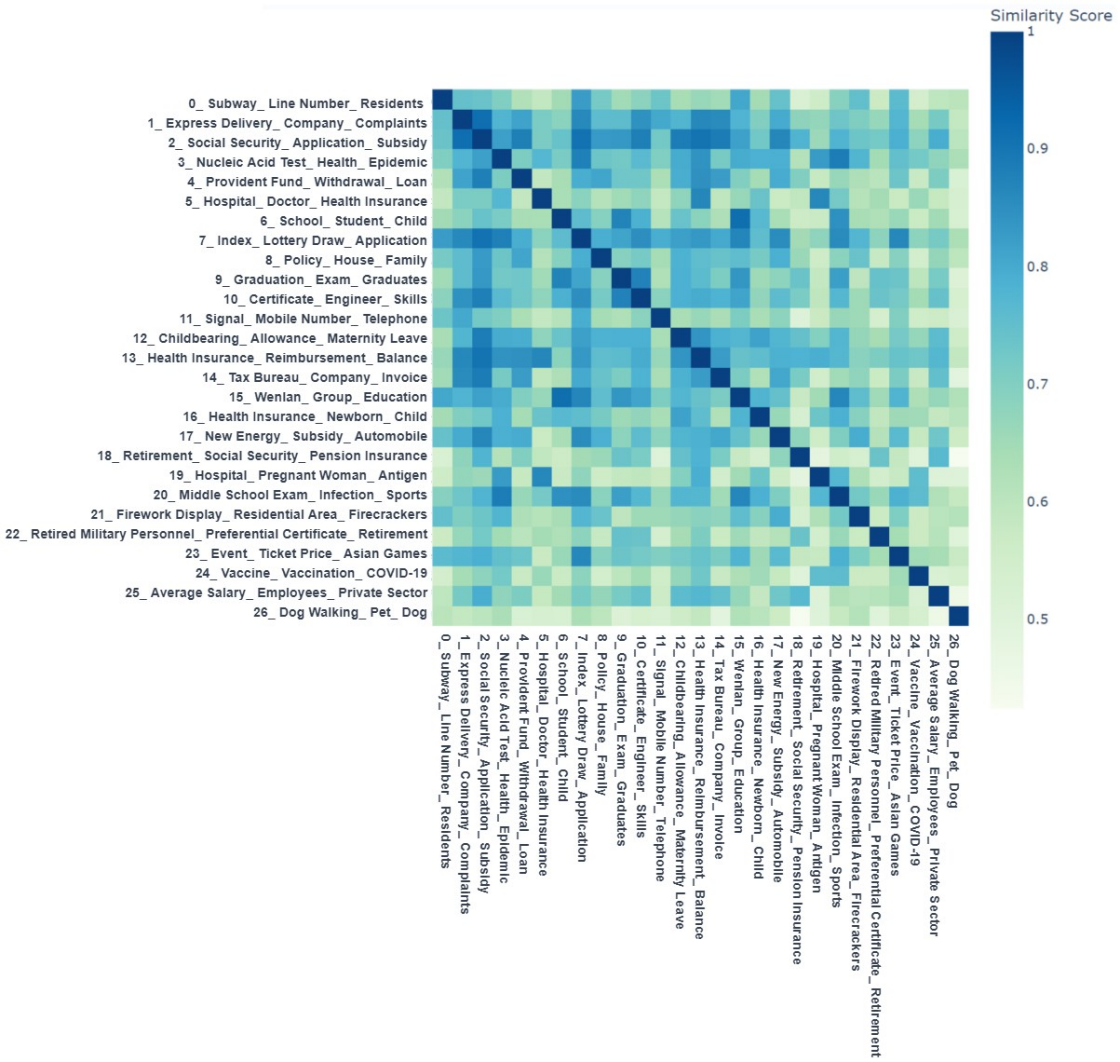
Secondary topic similarity matrix diagram. The figure displays the similarity between secondary topics. In the heatmap, the depth of the color represents the level of similarity: a darker color indicates a higher similarity, meaning the compared pair of topics are more similar in content; conversely, a lighter color signifies a lower similarity, indicating greater content differences between the topics being compared.

From the secondary topic distribution diagram (Fig 8), we discovered that some topics are very close to each other, and there is still room for further consolidation between topics in order to extract primary topics. The topic hierarchy diagram (Fig 12) generated a dendrogram to visualize the hierarchical clustering of the 27 topics, with topics of the same color possessing higher similarity. The topic similarity matrix diagram (Fig 13) quantitatively displays the pairwise similarity between each topic. Therefore, Figs 12 and 13 can be used as guides for topic merging.

**Fig 14:**
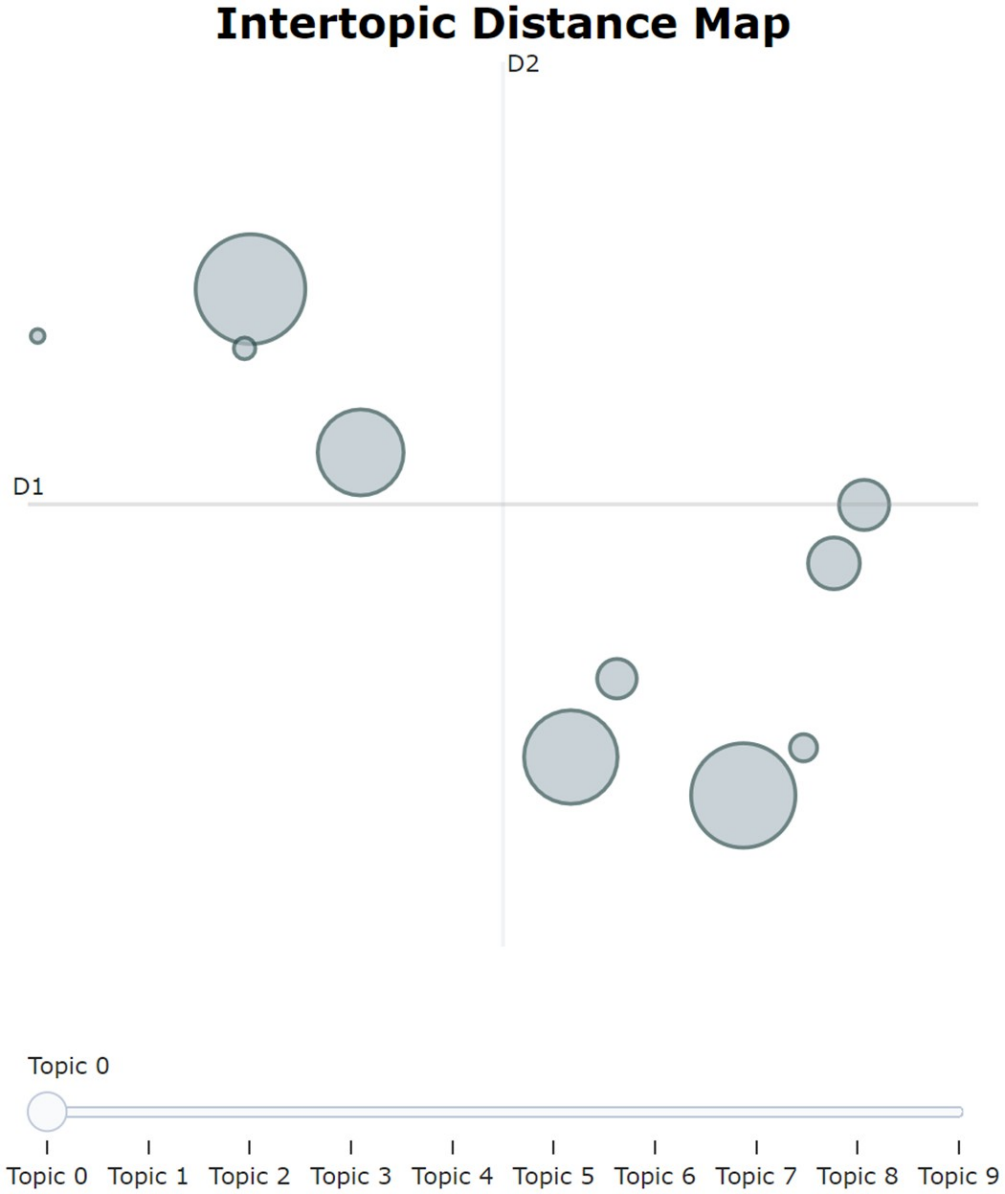
Primary topic distribution diagram. The figure displays the distribution of primary topics within a two-dimensional scaling space.

We employed the manual aggregation method in BERTopic based on the visualization results (Figs 12 and 13) and team analysis opinions to extract primary topics. Fig 13 shows that Topic 0 (Metro Travel) and Topic 7 (Vehicle Quota Acquisition) have a similarity of 0.82, while Topic 7 (Vehicle Quota Acquisition) and Topic 17 (New Energy Vehicle Policy) have a similarity of 0.86. In Fig 12, it is also recommended to merge Topics 7 and 17. From a professional knowledge dimension, Topics 0, 7, and 17 are all related to transportation and travel and can be merged into the same topic. Similarly, Topics 2 (Social Security Handling), 4 (Provident Fund Withdrawal and Loans), 12 (Maternity Benefits), etc., can also be merged into the same topic. After sequentially reviewing the structure of each topic, using BERTopic for manual topic merging and model updating, we finally determined 10 primary topics. The primary topic distribution diagram (Fig 14) shows that the distribution of each topic is relatively dispersed with small local overlaps, displaying a relatively ideal clustering effect.

**Fig 15:**
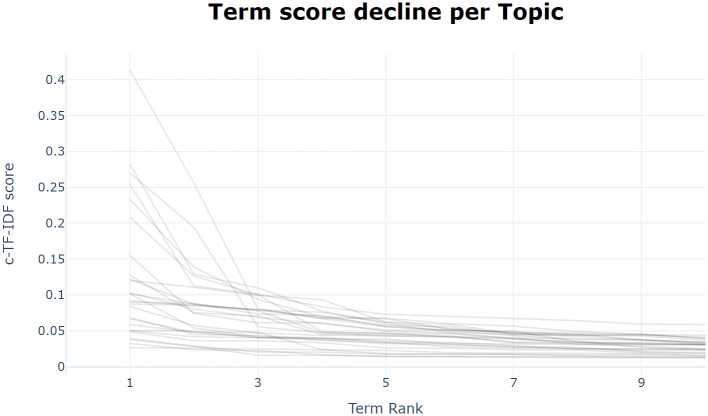
Secondary topic keyword contribution line chart. The chart depicts the trend of declining keyword contribution within various secondary topics. Each line in the chart represents a secondary topic, and the trajectory of the line illustrates the change in keyword contribution (c-TF-IDF score) with respect to the keyword ranking (Term Rank). The horizontal axis, "Term Rank," indicates the ranking of keywords within a topic, which is sorted based on their contribution. The vertical axis, "c-TF-IDF score," represents the contribution of keywords to the composition of a topic.

In the process of determining the names of each topic, we referred to the keywords within each topic. The composition of each topic consists of multiple words, where the higher the c-TF-IDF score for each word, the greater its contribution to the topic. The words for each topic are arranged in descending order according to their c-TF-IDF scores. Fig 15 displays the c-TF-IDF scores for 10 representative words for each topic with the horizontal axis representing the position number of the words within each topic and the vertical axis representing the c-TF-IDF scores. From this, it can be seen that the first three keywords of each topic can basically represent the main content of the topic. Based on this, the primary and secondary topics were named, and the results and main topic keywords are shown in Table 3.

**Table 3:** Topic-Keyword data output by the BERTopic model.

Primary Topic	Secondary Topic	Topic Keywords
Transportation	0. metro Travel	metro, Line Number, Residents, Travel, Community, Opening, Highway, metro Station, Vehicle, Suggestion
	7. Vehicle Quota Acquisition	Quota, Lottery, Application, Passenger Car, Review, Area, License Plate, Increment, Display, Time
	17. New Energy Vehicle Policy	New Energy, Subsidy, Car, Used Car, Quota, Policy, Emissions, National Standard V, Car Purchase, Vehicle
Consumer Rights Protection	1. Consumer Rights Protection	Express, Company, Complaint, Phone, Co., Ltd., Customer Service, Police Station, Service, Handle, Contract
	11. Communication Services	Signal, Phone Number, Phone, Telecom, Cellphone Number, Number, Community, Solve, Installation
Social Security	2. Social Security Handling	Social Security, Application, Subsidy, Payment, Handling, Provident Fund, Company, Talent, Place of Residence, Detail
	4. Provident Fund Withdrawal and Loans	Provident Fund, Withdrawal, Loan, Repayment, Handling, Account, Bank, Repay, Balance, House Purchase
	12. Maternity Benefits	Childbirth, Allowance, Maternity Leave, Reimbursement, Application, Policy, Social Security, Company, Claim, Payment
	18. Retirement and Pension Insurance	Retirement, Social Security, Pension Insurance, Payment, Pension, Pay, Handling, Personnel, Equated, Seniority
	22. Veterans’ Benefits	Veterans, Preferential Treatment Certificate, Retirement, Soldier, Preferential Treatment, Military, Free, Demobilized Soldier, Degree Upgrade, Policy
	25. Wages and Employee Rights	Average Salary, Employee, Private, Annual, Three Times, Publish, Society, Statistics Bureau, Unit, Salary
Medical Services and Security	3. Epidemic Prevention and Control	Nucleic Acid, Health, Epidemic, Yellow Code, Testing, Three Codes, Rules, Verification, Change, Department
	5. Medical Services	Hospital, Doctor, Health Insurance, Hospital Area, Related, Surgery, Inform, Visit, Affiliated, Outpatient
	13. Health Insurance Management	Health Insurance, Reimbursement, Balance, Account, Payment, Previous Years, Pay, Transfer, Mutual Aid, Display
	16. Child Health Insurance	Health Insurance, Newborn, Child, Kid, Children, Handling, Parents, Baby, Household Registration, Mutual Aid
	19. Medical Care for Special Groups During the Pandemic	Hospital, Pregnant Woman, Antigen, Pharmacy, Medicine, Epidemic, Nucleic Acid, Fever, Open, Policy
	20. Education and Pandemic	Middle School Entrance Examination, Infection, Physical Education, Children, Students, Epidemic, Examination, COVID-19, Cancellation, Schoo
	24. Vaccination	Vaccine, Inoculation, COVID-19, Household Registration, Nine-Valent, Age, Quadrivalent, Community Hospital, Appointment, Inhalation
Education	6. Education System	School, Student, Child, Teacher, Exam, Education, Parent, Major, Examinee, College
	15. Educational Institutions	Wenlan, Group, Education, Experimental School, Canal, Elementary School, Education Bureau, Agreement, Gongshu District, School Area
Employment and Career Development	9. Employment	Graduation, Exam, Graduates, Talent, Application, Registration, File, Subsidy, Undergraduate, Postgraduate
	10. Professional Qualifications and Certification	Certificate, Engineer, Skill, Application, Senior, Professional, Talent, Registration, Enterprise, Unit
Urban Living and Services	21. Urban Life and Safety Management	Fireworks, Community, Firecrackers, City, People, Firefighting, Air Conditioning, Life, Electricity, Ignition
	26. Pet Management	Walking the Dog, Pet, Doggie, Dog Raising, City, Civility, Dog Raising, Time, Dog License, Dog License
Real Estate Policy	8. Real Estate Policy	Policy, House, Family, Housing, Lottery, House Purchase, Loan, Talent, House, Social Security
Tax Management	14. Tax Management	Tax Bureau, Company, Invoice, Tax, Business, Tax Evasion, Merchant, Co., Ltd., Declaration, Report
Sports and Cultural Activities	23. Asian Games Events	Event, Ticket Price, Asian Games, Final, Ticket, Hosting, E-Sports, Directing, Watching the Game, Same Period

Analysis revealed that public demands can be divided into 10 primary topics and 27 secondary topics (Table 4). Out of 17,593 letters, 4,485 (25.49%) pertain to Topic A, which revolves around Transportation, encompassing secondary topics such as Metro Travel, Vehicle Quota Acquisition, New Energy Vehicle Policy, etc. Therefore, the Transportation topic related to daily commuting is the public’s primary concern. Ranking second is Social Security, labeled Topic B, with 4,057 (23.06%) letters encompassing Social Security Handling, Provident Fund Withdrawal and Loans, Maternity Benefits, Retirement and Pension Insurance, Veterans’ Benefits, Wages and Employee Rights. The development of the social security system directly affects people’s livelihood and welfare, and this topic has received widespread attention. Topic C, Medical Services and Security, accounts for 3,261(18.54%) of letters, including Epidemic Prevention and Control, Medical Services, Health Insurance Management, etc. This topic reflects people’s emphasis on health, especially against the backdrop of the COVID-19 pandemic, where medical services and security issues are particularly important. Topic D, Consumer Rights Protection, accounts for 2,752 (15.64%) letters, where the public mainly focuses on Express Delivery Services, Related Complaints, Contract Signing, Police Station Assistance, Signal and Communication Services, etc., expecting timely and effective solutions. According to the Pareto Principle, cumulative factors in the 0–80% range are the main factors [55]. Topics A, B, C, and D collectively account for 82.73% of correspondences, so these four topics can be considered the most significant public demands. Among other topics, Topics E, Employment and Career Development, and F, Education, mainly focus on issues in education, employment, and career development. Within the entire education system, the public is more concerned about education policies; relationships between teachers, parents, and students; school selection; education equity; service quality complaints; job hunting; professional qualification certification; etc. Topic G, Real Estate Policy, has also been a hot issue for the public. In recent years, local governments have successively introduced a series of policies such as purchase restrictions, loan restrictions, talent introduction, etc., to achieve stable and healthy development of the real estate market, with policies being adjusted at different stages. The public is more concerned about the latest housing policies, lottery policies, and specific requirements for loans and social security. Additionally, Topics H, Tax Management, and I, Urban Living and Services, are also hotspots for public attention. It is worth mentioning that the 19th Asian Games was held in Hangzhou in September 2023, and this event has become a focus of public attention and discussion.

**Table 4:** Topic information mined by the BERTopic algorithm (N = 17,593).

Primary and Secondary Topics	Quantity n (%)
**A Transportation (n = 4485, 25.49%)**	
0. metro Travel	3588 (20.39)
7. Vehicle Quota Acquisition	681 (3.87)
17. New Energy Vehicle Policy	216 (1.23)
**B Social Security (n = 4057, 23.06%)**	
2. Social Security Handling	2062 (11.67)
4. Provident Fund Withdrawal and Loans	1323 (7.52)
12. Maternity Benefits	330 (1.88)
18. Retirement and Pension Insurance	214 (1.22)
22. Veterans’ Benefits	73 (0.41)
25. Wages and Employee Rights	55 (0.31)
**C Medical Services and Securit (n = 3261, 18.54%)**	
3. Epidemic Prevention and Control	1380 (7.84)
5. Medical Services	988 (5.62)
13. Health Insurance Management	316 (1.80)
16. Child Health Insurance	221 (1.26)
19. Medical Care for Special Groups During the Pandemic	149 (0.85)
20. Education and Pandemic	138 (0.78)
24. Vaccination	69 (0.39)
**D Consumer Rights Protection (n = 2752, 15.64%)**	
1. Consumer Rights Protection	2371 (13.48)
11. Communication Services	381 (2.17)
**E Employment and Career Development (n = 999, 5.67%)**	
9. Employment	504 (2.86)
10. Professional Qualifications and Certification	495 (2.81)
**F Education (n = 938, 5.433%)**	
6. Education System	699 (3.97)
15. Educational Institutions	239 (1.36)
**G Real Estate Policy (n = 582, 3.31%)**	
8. Real Estate Policy	582 (3.31)
**H Tax Managemen (n = 274, 1.56%)**	
14. Tax Managemen	274 (1.56)
**I Urban Living and Services (n = 174, 0.99%)**	
21. Urban Life and Safety Management	127 (0.72)
26. Pet Management	47 (0.27)
**J Sports and Cultural Activities (n = 71, 0.4%)**	
23. Asian Games Events	71 (0.4)

## Discussion

### Principal findings

#### Public demands present diversified characteristics

Online government inquiries have gradually become the main channel for citizens to express their demands and make suggestions in modern society, and the public’s demands have shown diversified characteristics. The "People Call Me" platform involved in this study covers issues in various fields such as transportation, medical care, education, housing, rights protection, taxation, safety, etc. This also confirms that people’s demands for a better life are not only reflected in material life but also in democracy, rule of law, fairness, justice, safety, environment, and other diversified, multi-level, and multi-faceted requirements [56]. The emergence of this phenomenon reflects the new trend of communication between our government and citizens and reveals the positive outcomes of public participation in policy-making and social governance. A total of 522 government departments have actively responded to the public’s questions, further proving the platform’s usefulness, and showing the government departments’ attentive responsiveness to public opinions. The public’s reflections on issues ranging from suggestions for national policies (such as the "Double Reduction" policy) to complaints about dog leash problems can all receive corresponding attention and handling on this platform. This fully illustrates the important role of the online government inquiry platform in promoting government transparency, enhancing citizen participation, and improving governance efficiency. In addition, the study found that some demands are made in the form of collective complaints, such as the "affiliation issue of the Wenlan School District in the Gongshu District Canal New City Unit," which collected 35 complaints. A few researchers have also paid attention to this kind of issue, where citizens collectively amplify an issue to elicit a response from the government, and this conscious, strategic expression behavior is worth exploring in depth [57]. Overall, the online government inquiry platform has played an irreplaceable role in listening to public opinion, alleviating people’s difficulties, and gathering public’s wisdom. This is particularly evident in its effective detection and monitoring of emerging trends in mass public opinion and hot issues in social governance, providing intelligent support for the scientific formulation and implementation of public policies, and contributing to the realization of the main function of common prosperity for the general public [58].

#### Public demands focus on life security and rights protection

On the online government inquiry platform, Transportation, Social Security, Medical Services and Securit and Consumer Rights Protection have become the current primary demands. These demands not only reveal the core demands of society but also reflect the public’s deep concern for basic living quality and their own rights. Although the government’s response to these demands has been positive, it also reveals some challenges. For example, in terms of transportation, in the past two years, to better prepare for the Asian Games, Hangzhou has continuously accelerated the construction of the metro. As of August 1, 2023, the total mileage of Hangzhou’s metro network has reached 516 kilometers, forming a rail transit planning network that matches Hangzhou’s international development level. The "Hangzhou Comprehensive Transportation Special Plan (2021–2035)" released by the Hangzhou Municipal People’s Government in September 2021 also emphasizes that by 2035, the total mileage of Hangzhou’s metro traffic network will reach more than 1100 kilometers [59]. Although the government has invested a lot of resources in public transportation and infrastructure construction, there are still regions and populations that have not fully benefited. Among the transportation topics, there are 1214 letters related to the metro, and public demands focus on metro line construction and planning, complaints about metro service quality and attitude, adjustment demands for metro operations and shifts, coordination issues between metro and surrounding traffic, integration of metro and epidemic prevention measures, and disturbances caused by metro construction, etc. In terms of basic social security and medical protection, the government is also making continuous efforts. In recent years, Zhejiang Province has actively responded to the call of the Party Central Committee to "promote the modernization of the national governance system and governance capacity," taking the lead in initiating the "Run at Most Once" reform, which has produced positive social effects [60]. Similar measures are gradually being carried out in various fields and departments, such as Hangzhou’s public hospitals fully promoting the "Run at Most Once" reform and action, greatly optimizing and improving the patient’s medical process and experience [61]. However, the public still has a large number of issues such as provident fund extraction, social security payment, medical service complaints, wage arrears, labor disputes, etc. In addition, some niche demands, such as the service demands of special populations like the elderly, children, disabled people, international students, assistance for rare disease patients, work injury identification compensation, etc., deserve the attention and emphasis of the government and society.

Through the online government inquiry platform, the government can promptly understand the public’s core demands and control the direction of public opinion. At the same time, the timely response and quality of replies from various departments are particularly important. If the accountability for online public opinion is not timely or in place, it may easily trigger secondary public opinion and collective behavior, exacerbating the relationship between officials and the public [62]. According to this study, government departments were able to provide timely feedback on the public’s core demands, with 67.6% of the letters having received a response within 5 days and 93.5% of the letters having received feedback within 10 days. This also fully demonstrated the government’s high regard for the public’s core demands.

#### The key role of public participation in emergency management

The COVID-19 pandemic has been dubbed by the WHO as "the most severe global emergency health event" since its inception, posing unprecedented challenges to global public health governance and socio-economic development [63]. In this study, through the analysis of letters on an online governmental inquiry platform, we identified the public’s concerns and demands in the post-pandemic era regarding major public health events, particularly in pandemic management. From the sub-themes, #3 Pandemic Control, #19 Medical Treatment for Special Groups under the Pandemic, #20 Education and the Pandemic, and #24 Vaccination, we can clearly see the public’s urgent focus on pandemic testing, medical resources, educational impacts, and vaccination. These topics span from basic nucleic acid testing to the special needs of pregnant women and students, as well as detailed issues of vaccination, covering all aspects of pandemic management. Out of 1,736 letters related to the pandemic, 1,473 conveyed negative emotions, accounting for as much as 84.9%, highlighting the widespread concern about the pandemic. Notably, the average response cycles of Hangzhou Municipal Medical Security Bureau and Hangzhou Municipal Health Committee on the platform were 3.37 days and 2.65 days, respectively. Such timely responses not only reflect the government’s high attention to significant safety events but also help alleviate public anxiety, enhancing governmental credibility and trustworthiness among citizens. Since China’s government announced the easing of pandemic control policies in December 2022, public attention to the pandemic has significantly waned. The sharp decline from 307 letters in December 2022 to 51 in January 2023 reveals a close correlation between public demands and national policy changes. This not only underscores the sensitivity of the online governmental inquiry platform to policy shifts but also illustrates the intimate connection and mutual influence between government decisions and public demands, providing real-time feedback and direction for the government to further understand and meet the needs of the people.

In recent years, global climate change has led to frequent, widespread, intense, and concurrent extreme weather events such as heavy rainfall, heatwaves, and super typhoons, posing enormous challenges to urban governance in China and highlighting the urgent need to enhance preparedness, perceptiveness, and responsiveness [64]. Against this backdrop, timely perception and scientific response to public demands have become key. Through channels like the online governmental inquiry platform, the government can better understand the real-time needs of the public, thereby making more precise and targeted decisions. Whether in pandemic control or extreme weather events, this ability is crucial for safeguarding public life and property, and enhancing post-disaster resilience. This also emphasizes that the government must continue to strengthen communication and cooperation with the public in future urban governance and public safety management, responding to various emergencies in a scientific, humane, and efficient manner to ensure the safety and stability of the populace.

#### The successful attempt of BERTopic in the field of government affairs

The advanced BERTopic algorithm has been applied in various fields such as user feedback, employee surveys, speech perception, social media, IT service management, electronic health records, and more. It is also increasingly utilized in academic research, such as in the assessment of cancer health disparities [65], mining citizen emotions under sudden public health events [66], and evaluating impressions of tourist destinations [67]. In previous studies, there has been little integration of the BERTopic algorithm into the field of government affairs. This research represents an exploratory attempt of BERTopic in the field of government affairs. The study employed BERTopic for topic analysis and dynamic topic analysis. From the results of the topic analysis, the public’s hot demands are in the fields of transportation, social security, medical services and protection, and consumer rights protection; from the results of keyword frequency in statistical analysis, the hot words are related departments, housing provident fund, metro, social security, complaints, etc.; from the statistical analysis of the responding units, Hangzhou Metro Group Co., Ltd., Hangzhou Transportation Bureau, Hangzhou Housing Security and Real Estate Management Bureau, Hangzhou Housing Provident Fund Management Center, Hangzhou Medical Security Bureau, Hangzhou Housing Provident Fund Management Center are the units with the most letters. The results of the topic analysis and statistical analysis are basically consistent, and combined with manual assessment, it can be determined that the BERTopic algorithm has achieved good results in the field of government affairs, providing a more precise understanding of the semantics of online letters and identification of topics and more accurately capturing and analyzing the complex logic and relationships in government data. This study has verified the applicability of BERTopic in the field of government affairs. In the future, BERTopic can be applied to more government analysis scenarios, such as policy evaluation and public opinion analysis to provide more comprehensive insights.

### Limitations

This study conducted topic mining on the public’s letters of demand in an online government inquiry platform and statistically analyzed the time cycle of government departments’ responses. However, there was no in-depth exploration of the textual content of the responses, nor an evaluation of the quality of these responses. This approach would have allowed for a more comprehensive examination of the issues present in both online demands and government responses, thereby aiding the government in implementing improvements. Furthermore, future research may consider using the BERTopic online topic modeling program, which can adaptively update the model for incremental data, helping to capture and analyze the dynamic changes in public demands in real time, thus enabling the government to respond to social demands more sensitively.

## Conclusions

This study successfully introduced the advanced BERTopic algorithm into the field of government affairs. By conducting precise topic modeling and dynamic topic modeling on letters from the online government inquiry platform, it delved deeply into the core appeals and demands of the public. This could effectively assist the government in better listening to public opinions, respecting the will of the people, and aligning with the public sentiment. Furthermore, it could provide an important reference for optimizing public services and formulating scientific and reasonable policies. Additionally, this investigation illuminated the practicality and efficacy of the BERTopic algorithm within the realm of governmental operations. The results demonstrated that BERTopic has the potential to serve as an invaluable asset in governmental affairs, providing a sophisticated interpretation of the semantic content of online communications and facilitating the discernment of pertinent topics. This advancement enriches the existing knowledge base in the sphere of government management, bolstering the government’s capacity to comprehend and address the fundamental concerns and needs of the citizenry. Moreover, it paves the way for the algorithm’s application in a wider array of governmental analytical contexts, such as the evaluation of policies and the analysis of public sentiment, thereby bearing significant implications for the enhancement of contemporary governmental practices.

## Supporting information

S1 File Raw data. (XLSX)
